# From Sevagram to National Health Policy: Dr. Sushila Nayar’s Contributions to Rural Healthcare and Medical Education in India

**DOI:** 10.7759/cureus.76141

**Published:** 2024-12-21

**Authors:** Helan Rajan, S Johnson, Ram Prakash B U

**Affiliations:** 1 Otolaryngology-Head and Neck Surgery, Dr. Dnyandeo Yashwantrao Patil Medical College, Hospital and Research Centre, Dr. Dnyandeo Yashwantrao Patil Vidyapeeth (Deemed to be University), Pune, IND; 2 Community Medicine, Dr. Dnyandeo Yashwantrao Patil Medical College, Hospital and Research Centre, Dr. Dnyandeo Yashwantrao Patil Vidyapeeth (Deemed to be University), Pune, IND

**Keywords:** gandhian principles, healthcare in rural india, historical vignette, medical education, public policy in health, sevagram, sushila nayar

## Abstract

Dr. Sushila Nayar (1914-2001) was a pioneering figure in Indian public health whose work spanned from grassroots initiatives to national policy formation. This review article traces Dr. Nayar's remarkable journey and enduring impact on India's rural healthcare and medical education. Beginning with her early work at Sevagram alongside Mahatma Gandhi, the article explores how Dr. Nayar's experiences shaped her holistic approach to healthcare, emphasising preventive medicine and community involvement. Her innovative strategies in rural health delivery, including establishing the Mahatma Gandhi Institute of Medical Sciences in Sevagram, revolutionised medical education by integrating community-oriented learning. This review examines Dr. Nayar's pivotal role in shaping India's national health policies during her tenure as the Union Health Minister, highlighting her efforts in expanding rural health services and promoting indigenous systems of medicine. Furthermore, it analyses her contributions to maternal and child health, tuberculosis control, and leprosy eradication programs. It also discusses Dr. Nayar's challenges and how she overcame them, providing insights into her leadership style and vision. It comprehensively assesses Dr. Nayar's multifaceted legacy by examining archival materials, policy documents, and personal accounts. It concludes by reflecting on the relevance of her work in addressing contemporary healthcare challenges in India and other developing nations, underlining the lasting impact of her pioneering efforts in bridging the gap between public health, medical education, and national policy.

## Introduction and background

**Figure 1:**
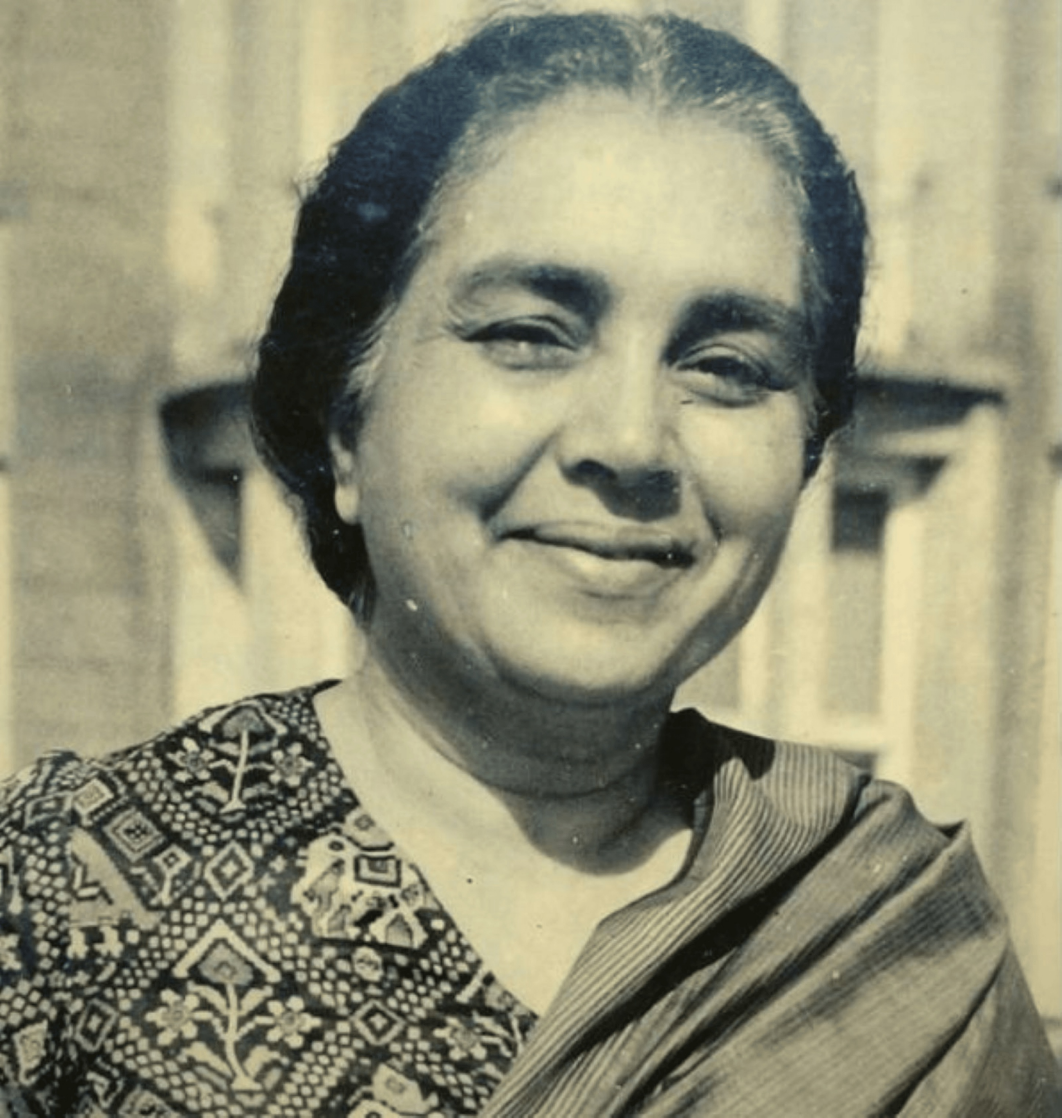
Dr. Sushila Nayar Source: Wikimedia Commons (Public domain)

Dr. Sushila Nayar (1914-2001) (Figure 1) was a pioneering figure in Indian medicine and public health and a close confidant and personal physician to Mahatma Gandhi. Her contributions as a freedom fighter, health minister, and advocate for healthcare reform were pivotal in shaping India’s post-independence healthcare landscape. Born into a family deeply committed to public service, Dr. Nayar became one of the leading voices in India’s struggle against infectious diseases like tuberculosis and leprosy, and her efforts significantly improved public health infrastructure, especially in rural India. Over her illustrious career, she harmonised Gandhian principles with modern medicine to create sustainable healthcare models that continue to influence public health practices in India today. This review explores her early life, her association with Gandhi, her public health initiatives, and her lasting legacy.

## Review

Early life and education

Sushila Nayar was born in 1914 in the undivided Punjab region in a family that strongly emphasised education and service. From a young age, she was influenced by her older brother, Pyarelal Nayar, who later became Mahatma Gandhi’s secretary and biographer. Her upbringing in such a socially-conscious household inspired her to pursue medicine as a tool for social change. Nayar earned her medical degree from Lady Hardinge Medical College in Delhi, one of the premier medical institutions in colonial India [1]. After Gandhi's assassination in 1948 in Delhi, Sushila Nayar went to the United States and earned two degrees in public health from the Johns Hopkins School of Public Health. Her earlier experience at Lady Hardinge Medical College had solidified her belief in the importance of accessible healthcare. This conviction would later drive her public health campaigns. During her medical training, she encountered the grim realities of poverty and disease in India’s urban and rural areas. These experiences further motivated her to dedicate her life to public service, merging her medical expertise with her passion for social justice.

Association with Mahatma Gandhi

Dr. Sushila Nayar’s close association with Mahatma Gandhi began in the 1930s when she joined Gandhi’s ashram at Sevagram. Her elder brother, Pyarelal Nayar, was the personal secretary of Mahatma Gandhi. She often visited Sabarmati Ashram to meet her brother. She met Mahatma Gandhi for the first time in 1929. Influenced by Mahatma Gandhi and his wife, Kasturba Gandhi, she started wearing Khadi. As a deeply spiritual and socially committed individual, in Gandhi’s philosophy of nonviolence and self-reliance, Nayar found a framework through which she could apply her medical knowledge to serve the people of India. Gandhi, too, found in her a compassionate and skilled physician whose medical interventions were rooted in a genuine commitment to alleviate suffering. She became Gandhi’s physician and caretaker during his fasts and political imprisonments. Her meticulous medical care is well-documented in Gandhi’s letters to her, which express his reliance on her expertise and dedication [2,3]. In a letter dated November 23, 1940, Gandhi wrote to Nayar with heartfelt gratitude for her care during an illness, marking the beginning of a lifelong professional and personal relationship between the two [3]. Nayar’s presence at Sevagram was not only medical; she became deeply involved in Gandhi’s broader mission for Indian independence. This partnership was most poignant during Gandhi’s final imprisonment in 1942, where Nayar continued to provide unwavering care. Her book 'Mahatma Gandhi’s Last Imprisonment: The Inside Story' offers an intimate look into this period, showcasing her unique position within Gandhi’s inner circle [4].

**Figure 2:**
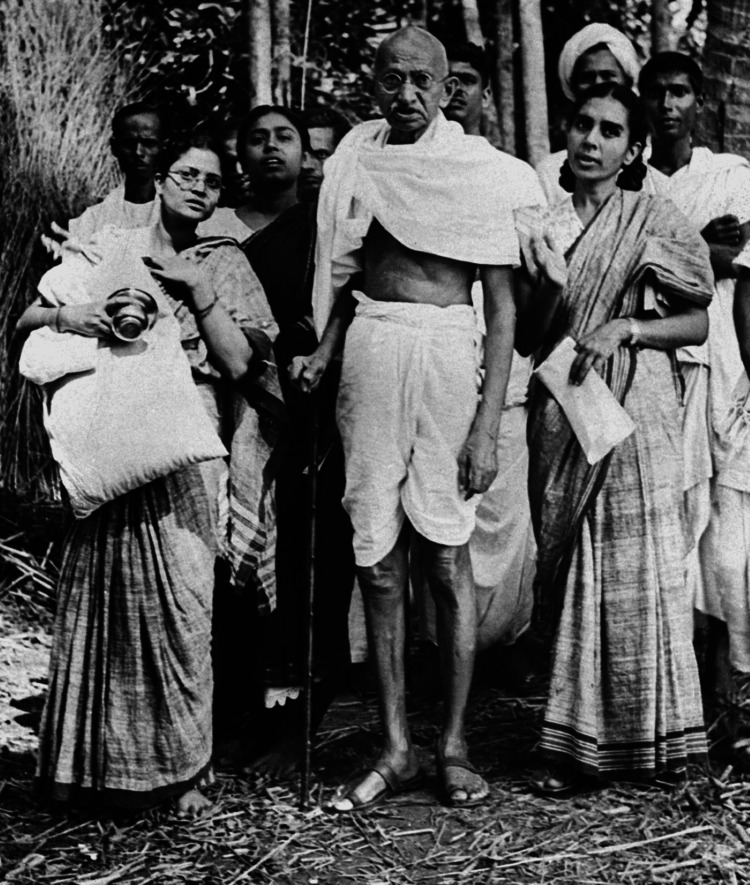
Dr. Sushila Nayar with Mahatma Gandhi and his grandniece Abha (on his right) Source: Wikimedia Commons (Public domain)

Contribution to public health

One of Dr. Nayar’s most important contributions to India was her role in public health. She saw the potential to apply Gandhian principles of simplicity, self-sufficiency, and service to the people in India’s healthcare system. After independence, Nayar dedicated herself to creating public health models that addressed the country’s most pressing medical challenges: leprosy, tuberculosis, and general access to medical care in rural areas. Her commitment to these causes was strengthened by her work at the Kasturba Hospital for Infectious Diseases, which became a centre for research and treatment under her leadership [5]. One of her most remarkable achievements was her role in developing anti-leprosy campaigns in the 1950s, an area that received little attention in mainstream medical circles at the time. Through awareness campaigns and treatment programmes, she helped reduce the social stigma attached to leprosy, transforming public attitudes towards the disease. This campaign is still cited as a model for tackling stigma around diseases in India [6]. Similarly, Nayar took on tuberculosis, a disease that had been devastating India’s rural and urban populations for decades. Her approach to tuberculosis treatment combined modern medicine with an emphasis on nutrition, sanitation, and patient education. Her contribution to this field was recognised nationally and aligned with Gandhi’s philosophy of prevention being better than cure [7]. Nayar’s influence extended beyond direct medical treatment. She played a crucial role in shaping the medical education system, and helped establish institutions that trained future generations of healthcare workers to follow her lead in focusing on preventive care and rural outreach. Her efforts in founding the Mahatma Gandhi Institute of Medical Sciences (MGIMS) in Sevagram aimed to address the vast disparities in access to healthcare in India [8].

Public health and Gandhian philosophy

Dr. Nayar’s public health work was profoundly influenced by her close association with Gandhi. She sought to apply Gandhian principles to medicine by focusing on simple living, preventive care, and integrating modern medicine with traditional practices. Nayar believed that public health solutions should address the immediate symptoms of the illness and tackle the root causes of disease. She saw this as being tied to poverty, poor sanitation, and lack of education [7]. Her approach combined scientific rigour with a deep empathy for the patients she served. This balance between the modern and the traditional, the scientific and the philosophical, made her healthcare models such as Community Health Model, Primary Healthcare Model, Holistic Health Approach, Integration of Traditional and Modern Medicine, and Healthcare Training and Education Model sustainable and relevant to India’s rural population. In Gandhi’s letters to Nayar, he repeatedly emphasised the importance of her medical work and encouraged her to expand her outreach to more communities [7]. Nayar’s success in combating leprosy and tuberculosis is primarily attributed to her ability to organise community-based health programmes focused on curing diseases and preventing their spread. She worked closely with volunteers, often drawing from Gandhi’s network of social workers, to implement widespread public health education campaigns. Her ability to mobilise local communities and integrate healthcare with social reform was one of her greatest strengths as a public health leader [5].

Political contributions and the freedom movement

While Dr. Nayar’s public health work is perhaps her most lasting contribution, her role in India’s independence movement is also significant. As an active participant in the Quit India Movement of 1942, she was more than just a bystander to India’s political struggles. Nayar’s dual role as a healthcare provider and political activist illustrates her commitment to the country’s welfare [5]. During the Quit India Movement, Nayar was imprisoned along with Gandhi and other key leaders. Her time in jail did not dampen her spirit or desire to serve the nation. After her release, she continued supporting Gandhi and the independence movement, providing medical care to the freedom fighters and civilians during the tumultuous final years of the British rule. After independence, Nayar transitioned from being a freedom fighter to a critical figure in the government of the newly-independent India. She served as the Health Minister of Delhi, where she continued to focus on issues of public health, sanitation, and medical education. She later became the Union Health Minister under Jawaharlal Nehru, India’s first Prime Minister, where she played a vital role in shaping India’s national health policies [10].

Founding of medical institutions

Dr. Sushila Nayar’s influence on medical education in India is most clearly seen through her work in establishing the MGIMS in Sevagram. Founded in 1969, MGIMS was designed to train doctors to serve India’s rural population, a demographic long neglected by the country’s healthcare system [11]. Nayar’s vision for MGIMS was rooted in the belief that medical students should be skilled in modern medicine and become socially-conscious individuals who understand the needs of the communities they served. MGIMS was one of the first institutions to incorporate community-based learning into its curriculum, requiring students to spend time in rural areas as a part of their training [9]. The institution quickly gained a reputation for producing highly-skilled doctors and was deeply committed to public service. MGIMS continues to train healthcare professionals focusing on rural healthcare, carrying forward Nayar’s legacy of service and social responsibility [8].

Lasting impact on public health and medicine

Dr. Nayar’s work in public health and medical education had a transformative impact on India’s healthcare system. Her focus on preventive care, community involvement, and integrating traditional and modern medical practices created a healthcare model that was effective and culturally relevant to India’s diverse population [9]. Her contributions to the eradication of leprosy and tuberculosis are particularly noteworthy. Through her innovative health campaigns and her leadership in building institutions like MGIMS, Nayar laid the groundwork for future generations of public health leaders in India. Her efforts were recognised nationally and internationally and her work in leprosy control, in particular, earned her accolades from public health organisations worldwide. Moreover, Dr. Nayar’s commitment to social justice and healthcare equity has inspired healthcare professionals in India and beyond. Her ability to blend Gandhian principles with modern medical practices remains a guiding light for those who seek to address the root causes of illness and poverty through compassionate, community-based care [6].

## Conclusions

Dr. Sushila Nayar’s life is a testament to the power of combining medical expertise with a deep sense of social responsibility. Her legacy, which spans the fields of public health, medical education, and political activism, is one of profound service to her country. As Gandhi’s trusted physician and a tireless advocate for public health, Nayar’s contributions helped shape India’s healthcare system during its formative years and beyond. Her work combating leprosy, tuberculosis, and the overall healthcare challenges facing rural India continues to be felt today through the institutions she founded and the policies she helped shape. MGIMS, in particular, stands as a lasting tribute to her vision for a more just and equitable healthcare system in India. In the years since her passing, Dr. Nayar’s legacy has inspired healthcare professionals, public health advocates, and social activists. Her ability to combine the ideals of Gandhian philosophy with modern medicine has created a unique and enduring model for how healthcare can serve as a tool for social change.
